# Understanding patterns of accumulation: Improving forecast-based decisions via nudging

**DOI:** 10.3758/s13421-024-01519-6

**Published:** 2024-01-25

**Authors:** Hatice Zülal Boz-Yılmaz, Aysecan Boduroglu

**Affiliations:** 1 Department of Psychology, Bogazici University, 34342 Bebek, Istanbul, Turkey; 2 Department of Psychology, Koc University, 34450 Sarıyer, Istanbul, Turkey

**Keywords:** Nudges, Accumulation, Top-down effects, Decision-making, Forecasting, Reasoning

## Abstract

In this study we investigated challenges associated with comprehension of graphical patterns of accumulation (Experiment 1) and how to improve accumulation-based reasoning via nudging (Experiment 2). On each trial participants were presented with two separate graphs, each depicting a linear, saturating, or exponential data trajectory. They were then asked to make a binary decision based on their forecasts of how these trends would evolve. Correct responses were associated with a focus on the rate of increase in graphs; incorrect responses were driven by prior knowledge and beliefs regarding the context and/or selective attention towards the early phases of the line trajectories. To encourage participants to think more critically and accurately about the presented data, in Experiment 2, participants completed a nudge phase: they either made a forecast about a near horizon or read particular values on the studied trajectories prior to making their decisions. Forecasting about how the studied trajectories would progress led to improvements in determining expected accumulation growth. Merely reading values on the existing trajectory did not lead to improvements in decision accuracy. We demonstrate that actively asking participants to make specific forecasts prior to making decisions based on the accumulation trajectories improves decision accuracy.

**Supplementary Information:**

The online version contains supplementary material available at 10.3758/s13421-024-01519-6.

During everyday cognition, we encounter (visually presented) data, and form opinions and decide on courses of action. Ideally during these encounters, we are expected to process the visually presented data patterns in an unbiased manner and make informed decisions. Decades of research has revealed that both bottom-up features of graphs, existing graph schemas, domain knowledge and expertise and even cultural expectations lead to biases in how we process various graphic displays (Glaser et al., 2019; Harvey & Reimers, 2013; Hohle & Teigen, 2015; Ji, Zhang, & Guo, 2008; Theocharis et al., 2019; Tumen & Boduroglu, 2022). Recent research has shown that these biases in graph processing are critical not only because they impact one’s personal risk assessment in medical, financial, and other similar critical domains (De’Bondt, 1993; Johnson & Slovic, 1995; Reinholtz et al., 2021; Sobolev & Harvey, 2016), they also impact support for public policies (Guy et al., 2013; Reinholtz et al., 2021). More recently, pandemic-related research revealed that simple graph characteristics (Romano et al., 2020) (e.g., whether the y-axis presents values in log or linear scale) influenced people’s judgments of the seriousness of the pandemic and policy endorsements. Similarly, difficulty understanding exponential growth led to underestimated forecasts of expected COVID-19 spread, reducing support for social distancing policies (Banerjee et al. 2021; Fansher et al., 2022; Lammers et al., 2020). These studies highlight the need to better understand the link between graph comprehension, processing biases, and data-based decisions.

Judgmental forecasting tasks typically require viewers to process trended or untrended series and then make forecasts about how the pattern would evolve in the upcoming periods leading to certain decisions. The limited literature on judgmental forecasting has nonetheless revealed numerous bottom-up (Theocharis et al., 2019) and top-down factors (Glaser et al., 2019) that play an important role in judgmental forecasting (Harvey & Reimers, 2013). The data (e.g., their trend shape) are one important factor that affects forecasting decisions (Newman & School, 2012; Webby & O’Connor, 1996). For instance, O’Connor et al. (1997) found that participants made trend damping forecasts in downward and upward sale trends. They interpreted this result as people's overall inclination for expecting economic data reversal. Tumen and Boduroglu (2022) noted that the graph format (whether the trend was presented as a bar vs. line graph) further interacted with the impact of trend shape. When the graphs depict exponential trajectories as opposed to more linear trends, people typically underestimate trend growth in their forecasts (Doerner, 1980; Fansher et al., 2022; Wagenaar & Sagaria, 1975); the tendency to generate underestimated forecasts is reduced when the same information is presented in tabular format and when the growth patterns are less exponential (i.e. more linear) (Fansher et al., 2022; Wagenaar & Sagaria, 1975). These studies highlight how format and trend trajectories impact forecasts.

Forecasts are also influenced by top-down factors. De’Bondt (1993) found that non-experts showed a greater tendency to follow the trend in their forecasts in stock-price data while experts’ forecasts were more prone to estimate reversal; an effect attributed to cyclical patterns in economical contexts (Lawrence & Makridakis, 1989). Culture is another important factor affecting forecasting processes of people (Ji, Zhang, & Guo, 2008; Li et al., 2021; Wang, Gould, & Hou, 2014). For instance, Ji et al. (2008) demonstrated that Canadian people had more of a tendency to buy stocks that had an increasing trend and sell stocks that had a decreasing trend, while the behavior of Chinese people appeared to be the opposite. This pattern suggested that Chinese and Canadian people were more likely to expect reversals and stability, respectively, a pattern consistent with broader definitions of holistic and analytic cognitive styles characterizing these two cultural groups.

The literature is clear on the impact of both bottom-up and top-down factors impacting forecasts. In our work, we wanted to investigate how people utilize forecasts as they make certain decisions. In the current research we specifically investigate the impact of trend types of forecast-based decisions. We particularly focus on decisions based on forecasts that require people to extract cumulative information from graphical depictions of unit data. Previous literature has repeatedly shown that people have a hard time understanding accumulation (Cronin et al., 2009; Dutt & Gonzalez, 2012; Guy et al., 2013; Korzilius et al., 2014; Newell et al., 2016; Sterman, 2008; Sterman & Sweeney, 2002, 2007); this limited understanding of accumulation has also led to difficulties understanding complex dynamic systems.^1^ Specifically, studies investigating stock-flow reasoning have revealed that contrary to expectations, graphical presentations of input and output do not necessarily improve accuracy of stock estimates (Guy et al., 2013; Newell et al., 2016). In these stock-flow studies, viewers had to integrate simultaneously presented inflow and outflow information. However, this necessitates that participants not only understand the accumulation from the inflow, but also reductions linked to the outflow to reach the correct stock estimate. However, the literature is limited in that we do not have a good understanding of whether people can simply understand the increase in stock merely by looking at accumulation trajectories. Therefore, in our work, we specifically chose to focus on accumulation in a system where there was no outflow. In the first experiment we investigated whether people could correctly identify which one of two daily patterns presented visually would reach a cumulative target status sooner. We presented viewers with a pair of schematic graphs that either have an exponential, saturating, or linear profile (see Fig. 1); to identify the correct option participants needed to make accurate forecasts. Previewing our results, we not only demonstrated challenges in this forecast-based binary decision, but we also showed that the pattern of results varied according to the content of the scenarios. In Experiment 2, we examined the effectiveness of a nudge to improve forecast-based decisions and reduce contextual influences on decisions.

**Fig. 1:**
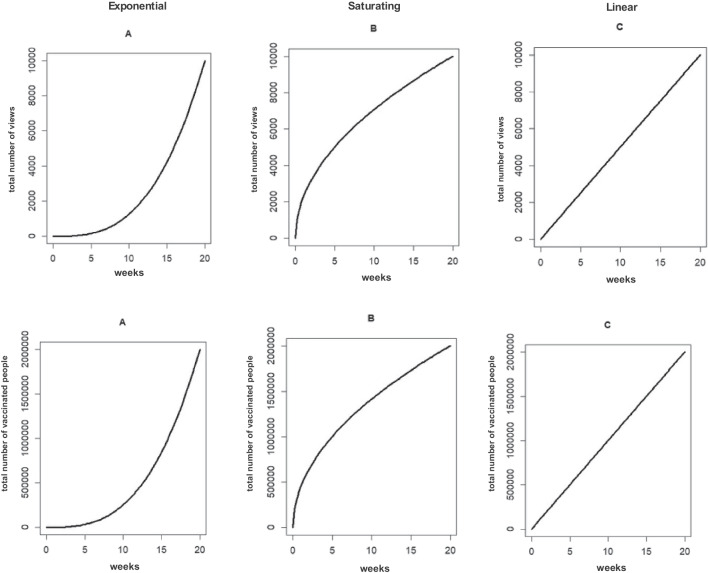
Example stimuli. The top panel shows the graphs used in the video context, while the bottom panel shows the graphs shown in the herd immunity context. In all graphs, the x-axis depicts weeks; the y-axis depicts the total number of video viewings and total number of vaccinated individuals, for the video and herd immunity conditions, respectively

## Experiment 1

The present study investigated forecast driven decisions based on data having different line trajectories. To solely focus on the impact of data trajectory shape on forecasts, in all cases we depicted trends reaching the same end value. The trajectories were either linearly increasing, exponentially increasing or saturating (see Fig. 1 for stimuli). To get some idea about generalizability of findings, participants were asked to make decisions given one of two different scenarios: in one, they were told that these trajectories represented the total number of views different music videos had in a set period of time; in the other case, they were told that the trajectories depicted the total number of vaccinated people in different countries, again within a set period of time. On each trial, participants were presented with a pair of trajectories, each on separate graphs and were asked the following question depending on the context. In the video context, they were asked to choose the video they thought had a promising track-record to becoming viral. In the vaccination case, they were told that 75% of the population needed to be vaccinated to achieve herd immunity and then were asked which of the two countries with the same population size that had vaccinated the same number of individuals was more likely to reach herd immunity earlier. In both contexts, participants were forced to commit to an explicit decision rather than indicate a preference over a continuous scale; this was done to mimic real-world contexts where people often need to choose one option over another. Participants were also asked to choose the graph from the pair that depicted either the higher rate of video viewing or higher rate of vaccination in the last week presented. We also asked participants to briefly explain their reasoning leading to their choice.

We expected the particular pairings of trend shapes to impact forecast-based decisions. Despite evidence showing exponential growth bias and underestimation of trend growth, we expected that people may nevertheless choose an exponential growing trend over the other options because the growth is easily visible. When saturating trends were coupled with linear ones, we thought choices may be impacted differently based on which segments of the trend participants attended to. The rapid increase of the trend at the start of a saturating graph could bias participants’ choice in favor of the saturating curve over the linear one, resulting in an erroneous response. Alternatively, the later segment of the saturating curve might give the impression that the trend is approaching a plateau. In that case, participants could choose the linear over the saturating graph; this would have led them to the correct option.^2^

We were mostly agnostic to the particular effects of context on decisions, nevertheless acknowledging the possibility that in the current pandemic context, decisions on herd immunity may in part be tainted by the particular media coverage participants had been exposed to.

### Method

#### Participants

We snowballed the sample via social media. Interested parties joined a Qualtrics link either on their mobile devices or computers. 1,081 individuals clicked on the link, but only 710 of them completed the main tasks. Based on the total time spent on the experiment we trimmed the top and bottom 5% from the data, leaving us with data from 640 participants (*M*_*age*_ = 25.67, *SD* = 5.57 years; 493 women, 138 men) (for detailed information, see Table S1 in Online Supplementary Material (OSM)). Two people did not report age information, and nine did not provide gender information. The majority of the participants in the sample were undergraduate (45%) and graduate students (22.5%), and 91.6 % of the non-student sample had at least a college or higher degree (for the detailed information, see the Table S2 in the OSM).

#### Materials

##### Background scenarios and tasks

There were two different background stories, video and herd immunity. Participants were randomly assigned to one of two contexts and were provided with the following scenarios (see below). In the video context, participants read the following scenario was about a music company planning to publish video songs on the online video streaming platform “VideoMe”:

Music videos of two different artists have been published from the account of the company. At the end of 20 weeks, each video was watched by 10,000 people in total. Those who manage this account want to advertise the videos in order to increase the number of views at the end of 20 weeks. A limited budget has been allocated for this goal. For this reason, the owners of the channel have decided to spend their money on a single video. They aim to choose the one with the highest potential and to ensure that it is watched by more and more people and even become viral. We will present you with line graphs showing the total number of views for these two videos over a span of weeks. Please assume advertisements will be effective. Answer the questions, keeping in mind the above-mentioned purpose.

In the herd immunity context, the scenario was about the countries trying to deal with a global pandemic. To achieve herd immunity, participants were told that each country should vaccinate 75% of its own population:

Two countries, each with a population of 10 million, have been vaccinating their own people for 20 weeks. At the end of 20 weeks, each country has vaccinated a total of 2 million people from its population, and none of them have yet achieved herd immunity. It is assumed that the vaccines are effective against the virus and there is no situation that will reduce their effectiveness. We will present you with line graphs showing the total number of vaccinated people in these two countries over a span of weeks. Answer the questions, keeping in mind the above-mentioned purpose.

Regardless of context, participants were randomly presented with a pair of graphs. Each graph presented a line trajectory; the pairing of the graphs per participant were randomly determined from a set of three possible combinations: linear versus exponential, linear versus saturating, exponential versus saturating. Except for the shape of the line trajectory, all other elements of graphs were the same within each context. In the video context, these graphs depicted the total number of views of a video over 20 weeks reaching 10,000 hits, and in the herd immunity context, the graphs depicted the total number of vaccinated people in a country over 20 weeks, reaching 2 million people (see Fig. 1). Participants saw the pair of graphs and the question on the same page. Graphs appeared horizontally (on PCs) or vertically (on phones). The locations of the graphs were counterbalanced across participants. Depending on the context, they were either asked to choose the video they think is likely to become viral with additional advertisement or the country that is going to reach herd immunity earlier. After participants indicated their decisions, we asked each one of them to provide an open-ended justification for their choice. To explore whether graph understanding predicted performance on the binary task, we presented the same graph pairs with a different question requiring them to read actual values on the presented data. They were asked to pick the graph that depicted a higher amount of video viewing/vaccinations in the last week.

##### Stimuli

Three different types of line graphs were created via R Statistical Software (v4.1.2; R Core Team 2021). The curve function was used to create exponentially increasing, linearly increasing and saturating line graphs. In each context, all lines reached the same value, 10,000 in the video context and 2 million in the herd-immunity context. We had to specify different functions to achieve the exact shape across the two contexts because of the variation in the y-axis range. The x-axis was the same for all graphs, ranging from 0 to 20 and denoted time passed in weeks. The function of the exponentially increasing graph was y = 1.25*x^3 in the video context and y = 250*x^3 in the herd-immunity context. The function of the linearly increasing graph was y = 500*x in the video context and 100,000*x in the herd-immunity context. The function of saturating graph was y = 2236.0679775*x^0.5 in the video context and 447213.5955*x^0.5 in the herd-immunity context.

##### Demographics and individual difference questions

We collected information about age, gender, and education. We also collected a number of exploratory measures. Participants reported whether they were a student (undergraduate/graduate) and whether they were a Bogazici University student/alumnus. Student participants were asked to indicate their majors, the year they were in, and the total number of mathematics courses they took during college (from 0 to 4 or more).

##### The comprehensive thinking styles questionnaire

To evaluate the thinking tendencies of participants, The Comprehensive Thinking Styles Questionnaire (Newton et al., 2021) was administrated. There are four sub-scales in this inventory which are named Actively Open-minded Thinking, Close-minded Thinking, Preference for Intuitive Thinking, and Preference for Effortful Thinking. We only used Preference for Intuitive Thinking and Preference for Effortful Thinking sub-scales, each including six items. All 12 questions were translated and back translated by undergraduate psychology students and verified by a professional translator. Participants were required to give answers over a six-point Likert scale ranging from 1 (*strongly disagree*) to 6 (*strongly agree*).

#### Design

In this experiment, a between subject design was used with the context (video and herd immunity) and graph pairs (exponential vs linear, linear vs saturating, and saturating vs exponential) as the two factors. Thus, there were six groups of participants and each person completed a single trial. Table 1 displays the number of participants in each group.

**Table 1:** The number of participants in each group

	Graph pairs		
Contexts	Exponential - saturating	Exponential - linear	Saturating - linear
Video	115	110	121
Herd immunity	105	93	96

#### Procedure

After participants signed the online consent form, we presented them a brief description of the experiment protocol. Participants were randomly assigned to video or herd immunity conditions. After reading the background scenario, they were randomly presented with a graph pair (linear vs. exponential, linear vs. saturated, exponential vs. saturated), they then completed the binary decision task, provided justification for their decision and then completed the graph understanding phase. Finally, they completed demographic and individual differences questions.

### Results

We coded all decisions as either correct or incorrect. Correct choices on the binary decision task were determined based on the mathematical projection of the presented trends. Because the endpoints presented in all graphs were identical, the growth progression suggested that the country/video that had the exponential trajectory regardless of what it was paired with, was the correct option. For the linear-saturating pair, the country/video who had the linear trajectory was the correct option.

To explore^3^ whether accuracy was different across graph pairs (exponential vs. linear, linear vs. saturating, and saturating vs. exponential) or across different contexts (video context and herd immune context), we conducted chi square analyses. For the binary decision task, there was no significant difference across the video (61.8%) and the herd immunity contexts (62.9%), ^*2*^ (1*, N* = 640) = .078, *p* = .78, *Cramer’s V* = .011. Also, accuracy was similar across graph pairs: exponential-saturating (64.1%), exponential-linear (66.5%), and linear-saturating graph pairs (56.7%), χ^*2*^ (2*, N* = 640) = 4.74, *p* = .093, *Cramer’s V* = .086.

**Fig. 2:**
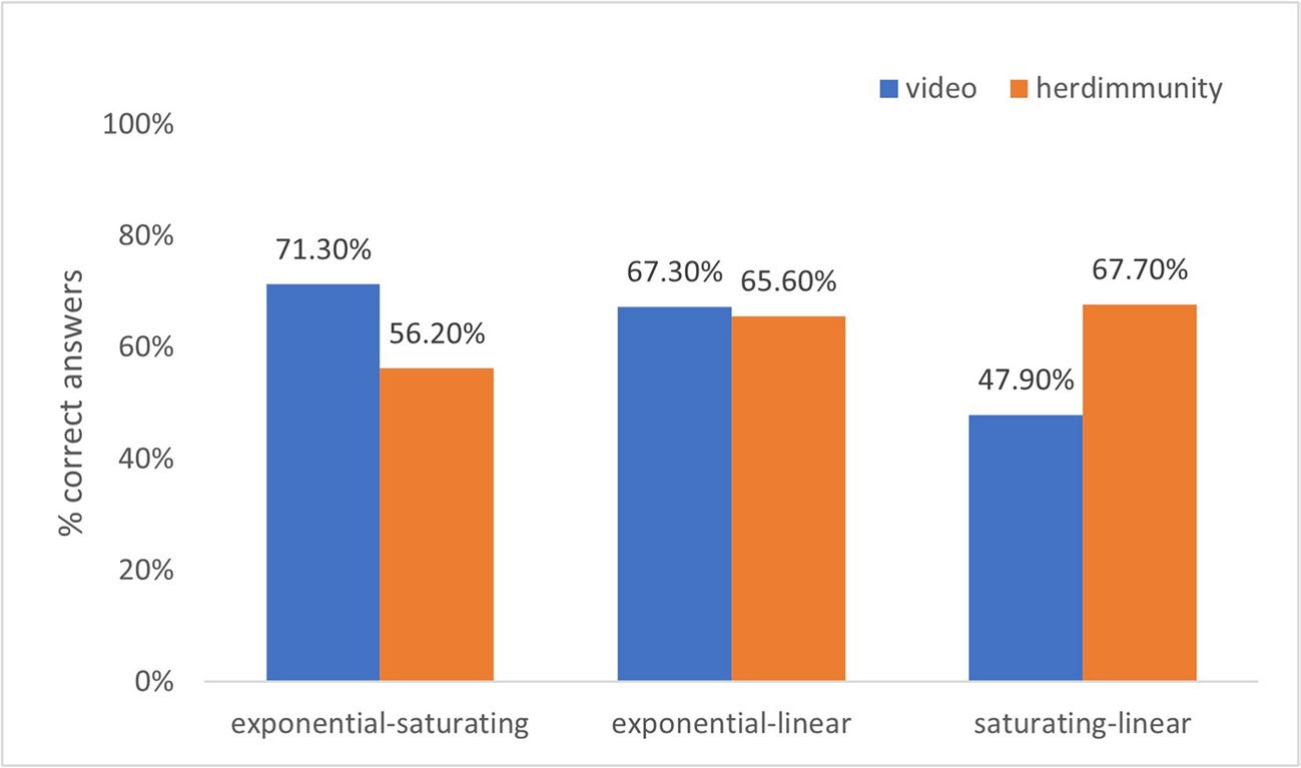
The distributions of Binary Decision Task’s accurate answers across graph pairs for each context

When we separately investigated the effect of graph pairs on each context, we found that in the herd immunity context, there was no effect of graph pairs, χ^*2*^ (2*, N* = 294) = 3.27, *p* = .195, *Cramer’s V* = .10; but in the video context, seeing different graph pairs significantly affected accuracy, χ^*2*^ (2*, N* = 346) = 15.67, *p* = < .001, *Cramer’s V* = .213. Specifically, in the saturating-linear pair, participants were less likely to identify the correct option (see Fig. 2).

We also analyzed the effect of context on each graph pair. When exponentially increasing and saturating graphs were presented, the correct answers of participants were significantly higher in the video context compared to the herd immunity context, χ^*2*^ (1*, N* = 220) = 5.45, *p* = .02, *Cramer’s V* = .16. When saturating and linearly increasing graphs were compared, participants who were in herd immunity context gave significantly more correct answers than participants in the other condition, χ^*2*^ (1*, N* = 217) = 8.52, *p* = .004, *Cramer’s V* = .198. On the other hand, when comparing exponentially and linearly increasing graphs, there was no significant difference in the answer across context, χ^*2*^ (1*, N* = 203) = .064, *p* = .80, *Cramer’s V* = .018 (Fig. 2)*.*

**Fig. 3:**
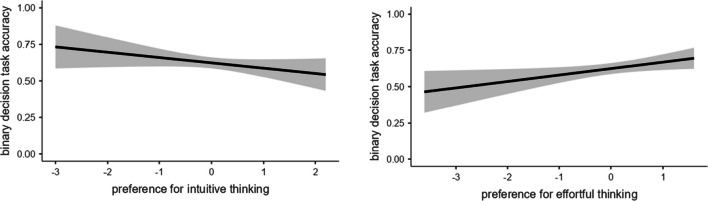
Binary decision task accuracy’s relationship with the Preference for Intuitive Thinking and Effortful Thinking

Point biserial correlations were computed to observe whether Preference for Intuitive Thinking and the Preference for Effortful Thinking were related to binary decision task accuracy. There was a weak but significant positive correlation between preference for effortful thinking and binary decision task accuracy, *r*_*pb*_ = .089, *n* = 640, *p = .*024. However, the relationship between the Preference for Intuitive Thinking and the task accuracy was not significant, *r*_*pb*_ = -.06, *n* = 640, *p* = .132 (Fig. 3).

#### Justifications for decisions

After completing the first task, participants were asked to provide justifications for their choice in a few sentences; 604 out of a total of 640 participants provided justifications for their choices. Two coders (an undergraduate lab member and HZB) identified six major categories by inspecting the first 40 participants’ responses. Afterwards, they separately coded the remaining responses (interrater reliability Kappa = .712, p < .001). Inconsistencies were resolved via discussion. Responses that mentioned the rate of increase, the derivative, the slope of the curve, exponential increase, and so on were coded as a “rate of increase” response. If responses emphasized the first or middle parts of the graph, these participants were categorized into the “focusing on first/middle parts of the graph” category. If participants referred to the linearity, linear increase, regularity, these responses were coded as “linearity” responses. If participants reported that they answered just by looking at the graph without giving any detail, we coded these responses into the “graph” category. When justifications included top-down information and reflected prior knowledge and/or beliefs, such as “it is better for the government to start vaccinating its people early than to vaccinate its people late, but in an intense schedule,” answers were coded as belonging to the “top-down” category. Finally, there was an “other” category for justifications that could not be categorized into any of the specified five categories. As can be seen in Table 2, more than half the participants (60.09%), gave justifications referring to the rate of increase in the trajectories; almost all of those (94.6%) correctly identified the right option in the binary decision task. Participants who focused on the first/middle parts of the trajectory, relied on top-down information, or linearity, were more likely to be mistaken.

**Table 2:** Open-ended categories according to binary decision task answers

Binary decision task			
Open-ended categories	False	True	Total
Rate of increase	20 5.4 %	348 94.6 %	368 60.9 %
Focusing on first/middle parts of the graph	80 97.6 %	2 2.4 %	82 13.6 %
Graph	6 60 %	4 40 %	10 1.7 %
Top-down	68 80 %	17 20%	85 14.1 %
Linearity	40 88.9 %	5 11.1 %	45 7.5 %
Others	8 57.1 %	6 42.9 %	14 2.3 %
Total	222	382	604
	36.8 %	63.2 %	

The table provides information relating the proportions of accurate responses for each justification category in the binary decision task. The percentage values in the far-right column show the % of total answers in each category and the false and true columns link the reported justification to task accuracy

To determine whether cognitive style was linked to task accuracy, we computed correlations between preference for intuitive thinking, preference for effortful thinking and task accuracy within each decision justification category. We found only one significant relationship. In the rate of increase category, there was a weak but significant positive correlation between preference for effortful thinking and binary decision task accuracy, *r*_pb_ = .136, *n* = 368, *p* = .009.

We also conducted exploratory analyses involving key demographic variables such as gender, education level and number of previously taken mathematics courses However, none of these correlations were significant, all *p* > .05.

### Discussion

In Experiment 1, we demonstrated that accuracy in the forecast-based binary decision task was often linked to justifications about the rate of increase in the line trajectories and was similar for both contexts and across different graph types. When people focused on early and middle parts of the trajectories, this led people into producing inaccurate decisions. A closer inspection of the data for each graph pair separately for the two contexts revealed meaningful differences across conditions. For instance, in the video context condition, the accuracy was significantly lower for the saturating-linear pair; performance was similar across graph pairs for the herd immunity condition. When performance was compared for the same graph pairs across the two contexts, in the saturating-linear and the saturating-exponential conditions, the accuracy patterns swapped. While our data does not allow us to definitely reveal why these differences emerge, we believe that these differences may be due to context-driven strategies impacting performance. It is also possible that context may also be exerting its influence via impacting certain stimulus characteristics. Even though we kept the shape of the graph pairs identical across the video and the herd conditions, the context necessitated that y-axis depicted different ranges and with different steps of increments. Also, in the video context, there were no explicit criteria stated whereas in the herd immunity context, we provided a target (75% of the population). These types of stimuli and instructional differences may have contributed to the differences observed across the two conditions.

## Experiment 2

In Experiment 1, we examined whether the answers of participants were affected by context and different pairs of graph trajectories. The results revealed that except for the exponential-linear graph pair, in the two other conditions (exponential-saturating and linear-saturating graph pairs), context played a key role in participants’ responses. Also, coding of open-ended justifications revealed that participants correctly responded if they focused on the rate of increase (94.6% of correct answers) in the data, and incorrectly responded if they used top-down (20% of correct answers) or selective attention strategies focusing at the beginning of line (2.4% correct answers) trajectories. We wanted participants to focus more on the data pattern rather than other top-down factors when responding in the binary decision task. In Experiment 2, we wanted to encourage participants to think in a more data-driven way and eliminate contextual influences. To this end, we tested the effectiveness of two separate nudges preceding the decision task.

Nudges are behavioral interventions that typically facilitate reasoning and they may vary in transparency and relation to the main task (Kozyreva et al., 2020). We specifically designed two nudges that would direct attention to the presented data trajectories. In the graph reading case, we specifically asked participants to report particular values on the already presented trend. We thought that this might help them focus on the rate of increase in the trends presented, a factor we had earlier identified to be associated with accurate decisions. Even though the graph reading nudge would lead to active engagement with the data presented, it would not directly be informative for the decision task because it does not guide people to focus on the progression of the cumulative pattern. This condition was therefore included as an active control. As an alternative, we also designed a forecasting nudge: we asked participants to make forecasts about how the trajectory would progress, but they were asked to consider relatively closer horizons than the expected decision targets. Experiment 2 was similar to Experiment 1 in many regards, except for the introduction of nudges that preceded the binary decision task and the exclusion of the exponential and linear graph pair since in Experiment 1 there were no context effects in that condition.

### Method

#### Participants

Experiment 2 was also conducted online via Qualtrics, and participants completed the experiment using either their mobile devices or computers. Eight hundred and twenty-three participants were recruited via snowballing, but not all participants completed the study leaving us with data from 605 participants. As in Experiment 1, we excluded the participants who spent either too little or too much time on the task compared to the remaining sample, using the top and bottom 5% criteria on total time spent (for the detailed information, see Table S3 in the OSM). This led to the elimination of 60 participants; analyses were carried out on data from 545 (*M*_*age*_ = 27.26, *SD* = 8.14 years) participants. While 369 of participants (*M*_*age*_ = 25.5, *SD* = 6.24 years) were women, 131 of participants were men (*M*_*age*_ = 32.19, *SD* = 10.6 years). Almost everyone who participated was a student or had at least a college degree (for the detailed information, see the Table S4 in the OSM).

#### Materials

In Experiment 2, we used the same binary task as Experiment 1 with the following exceptions. Because there were no differences across contexts (herd immunity vs. viral video) for the exponential-linear pair, we removed those subsets of trials. Critically, the main difference in Experiment 2 was the introduction of a secondary task preceding the main binary decision task. There were three between-subject conditions. We had a no-nudge condition to act as a baseline control, in which we replicated the general protocol from Experiment 1. Both in the graph reading and the forecasting nudge conditions, participants were asked to initially respond to questions related to the graph pair subsequently presented as part of the binary decision task. The graph reading condition was included in the study as an active control to ensure that participants would spend time, processing the graph pair as in the forecasting nudge condition. For both conditions, participants were presented with short introductory texts. In both cases they were asked to make a response which required them to read a particular value on the data presented (graph reading) or forecast a value (forecasting nudge). Please see below for the instructions (translated from the Turkish) for the herd immunity and the viral video conditions, respectively:

You will soon be shown graphs displaying the total number of people vaccinated over a 20-week period in two different countries. Answer the questions taking into consideration the graphs demonstrating the total number of values. Pay attention to the specific values reported on the y-axis. When you are ready, you can start the experiment by clicking the continue button.

You will soon be shown graphs showing the total number of views of two separate videos over a 20-week period. Answer the questions taking into consideration the graphs demonstrating the total number of values. Pay attention to the specific values reported on the y-axis. When you are ready, you can start the experiment by clicking the continue button.

**Fig. 4:**
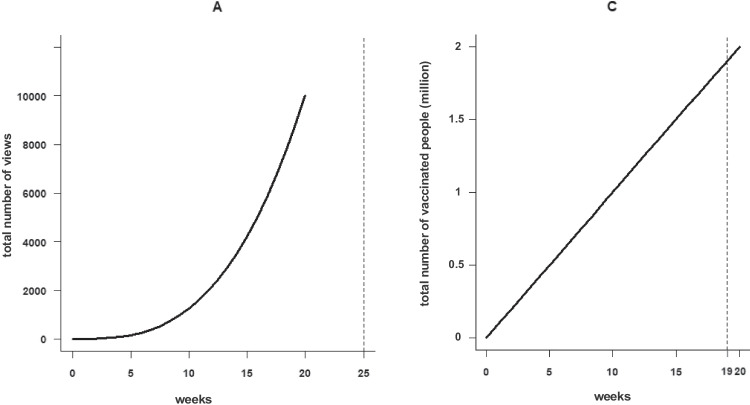
Forecasting and graph reading nudge displays. Example displays presenting what participants saw during the forecasting nudge (left, video context) and the graph reading nudge (right, herd immunity context). During the nudge phase, participants were presented with both graphs that they would subsequently be presented for the main task. In all graphs, the x-axis depicted weeks; the y-axis depicted the total number of video viewings or total number of vaccinated individuals, respectively. The y axis numbers on the graphs in the herd immunity condition are displayed in millions (for the other displays see Figs. S1 and S2 in the Online Supplementary Material)

In the forecasting nudge condition two graphs which were also presented in the following binary decision task, were displayed on separate pages. These pairs consisted of line graphs depicting either a saturating-linear or a saturating-exponential trend. The order of the graphs was counterbalanced. Each graph had a dotted vertical line *on week 25* and participants were asked the total number of views/people vaccinated *at week 25*. In the graph reading nudge condition, the only difference compared to the forecasting nudge condition was the place of the dotted line; the vertical dotted line was displayed at the 19th week (i.e., within the trend shown) and the question was: What is the total number of views/people vaccinated at the end of the 19th week? Participants reported their answers by using a slider. For the forecasting nudge, the values on the slider in the herd immunity group ranged from 0 to 4 million, while in the video group they were between 0 and 20,000. For the graph reading nudge, the values on the slider in the herd immunity group ranged from 0 to 2 million, while in the video group they were between 0 and 10,000 (see Fig. 4).

In Experiment 2, participants also completed the same demographics questions as in Experiment 1 and were in addition asked whether they had participated in Experiment 1. Only 11% of participants mentioned taking part in Experiment 1. Since there were nearly 6 months between the two experiments, we did not exclude those participants. In Experiment 2 we did not include the graph understanding task and the Comprehensive Thinking Style questions because responses on these correlated at best weakly with the binary decision task.

#### Design

In Experiment 2, a between subject design was used with the context (video and herd immunity), graph pairs (exponential vs linear and saturating vs exponential) and nudge (no nudge, graph reading nudge, and forecasting nudge) as the three factors. Thus, there were six groups of participants. Table 3 displays the number of participants in each group.

**Table 3:** The number of participants in each group

Nudges				
Contexts	Conditions	No nudge	Graph reading	Forecasting
Video	Exponential - saturating	50	46	45
	Saturating - linear	51	44	44
Herd immunity	Exponential - saturating	40	50	42
	Saturating - linear	42	46	45

#### Procedure

After providing consent, participants were presented with a brief description of the general protocol of the study. Then, they were randomly assigned to one of the three conditions (forecasting, graph reading or no-nudge group) evenly across the two contexts (herd immunity or viral video). Graph presentations were counterbalanced in both the graph reading and the forecasting nudge conditions. Afterwards, they were presented with the video or herd immunity scenarios and were asked to complete the binary decision task and provide justifications for their choices. In the no-nudge condition, participants directly completed the binary decision task. The study ended after participants responded to the demographic questions.

### Results

Our primary dependent variable was the accuracy on the binary decision task and the main independent variables were the context of the graphs (video or herd immunity), type of graph pairs (exponential vs saturating and saturating vs linear), and the nudge conditions (no nudge, graph reading nudge and forecasting nudge). We ran Chi-square tests to determine whether the accuracy in the binary decision task was impacted by the different contexts and graph types. The breakdown for all conditions across contexts is presented in the supplementary text (see OSM Table S5).

**Fig. 5:**
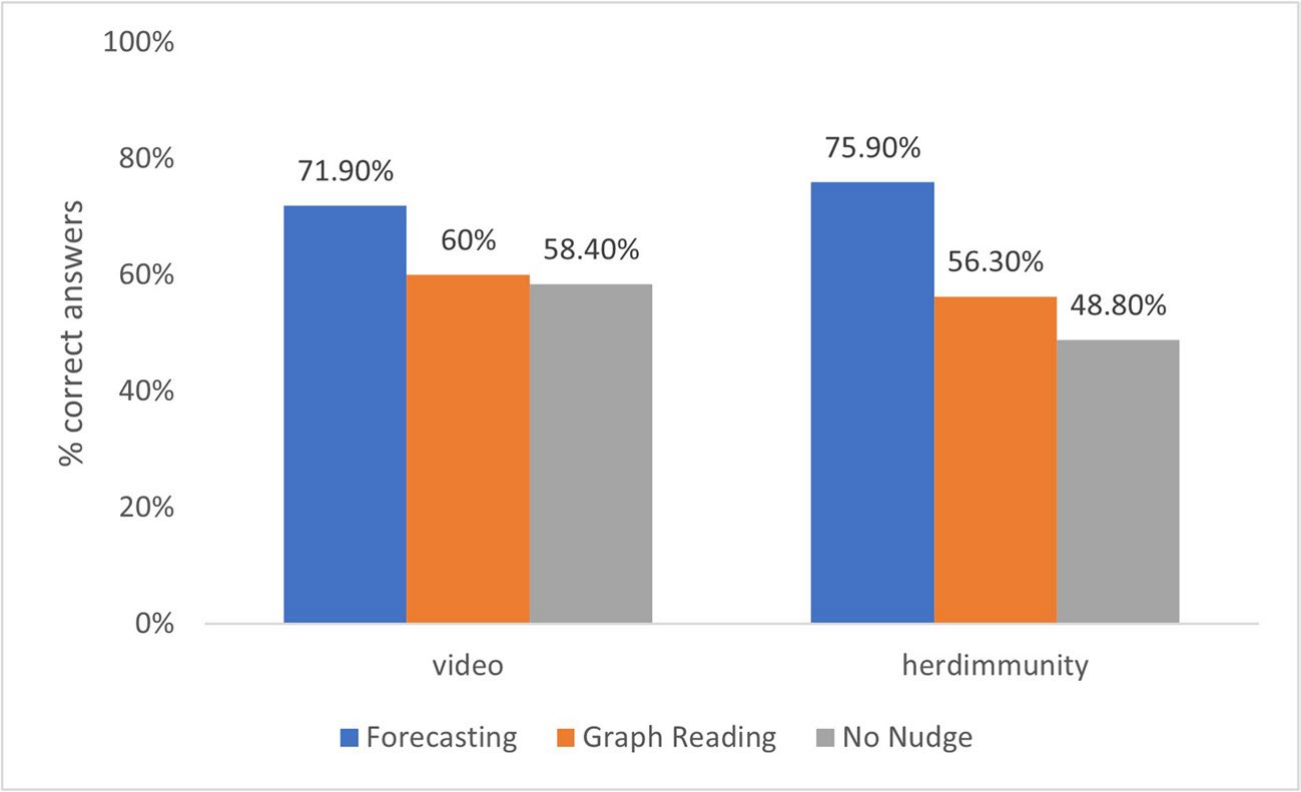
The distributions of Binary Decision Task’s accurate answers across nudge conditions in each context

To test whether there was an effect of nudges on accuracy on the binary decision task, we performed a chi square analysis (see Fig. 5). Accuracy in the forecasting nudge condition was considerably higher than in the no-nudge conditions for both the herd immunity χ^*2*^ (1*, N* = 169) = 13.241, *p* < .001, *Cramer’s V* = .28. and the video condition, χ^*2*^ (1*, N* = 190) = 3.774, *p* = .052, *Cramer’s V* = .141. We also compared the answers in the forecasting nudge with the graph reading condition. While participants were more accurate in the forecasting nudge than the graph reading condition in the herd immunity context, χ^*2*^ (1*, N* = 183) = 7.776, *p* = .005, *Cramer’s V* = .206, in the video context, these two conditions did not significantly differ, χ^*2*^ (1*, N* = 179) = 2.826, *p* = .093, *Cramer’s V* = .126. There were no differences in accuracy across the graph reading and no-nudge conditions for neither the herd immunity, χ^*2*^ (1*, N* = 178) = .99, *p* = .32, *Cramer’s V* = .075 nor the video contexts, χ^*2*^ (1*, N* = 191) = .049, *p* = .81, *Cramer’s V* = .016.

**Fig. 6:**
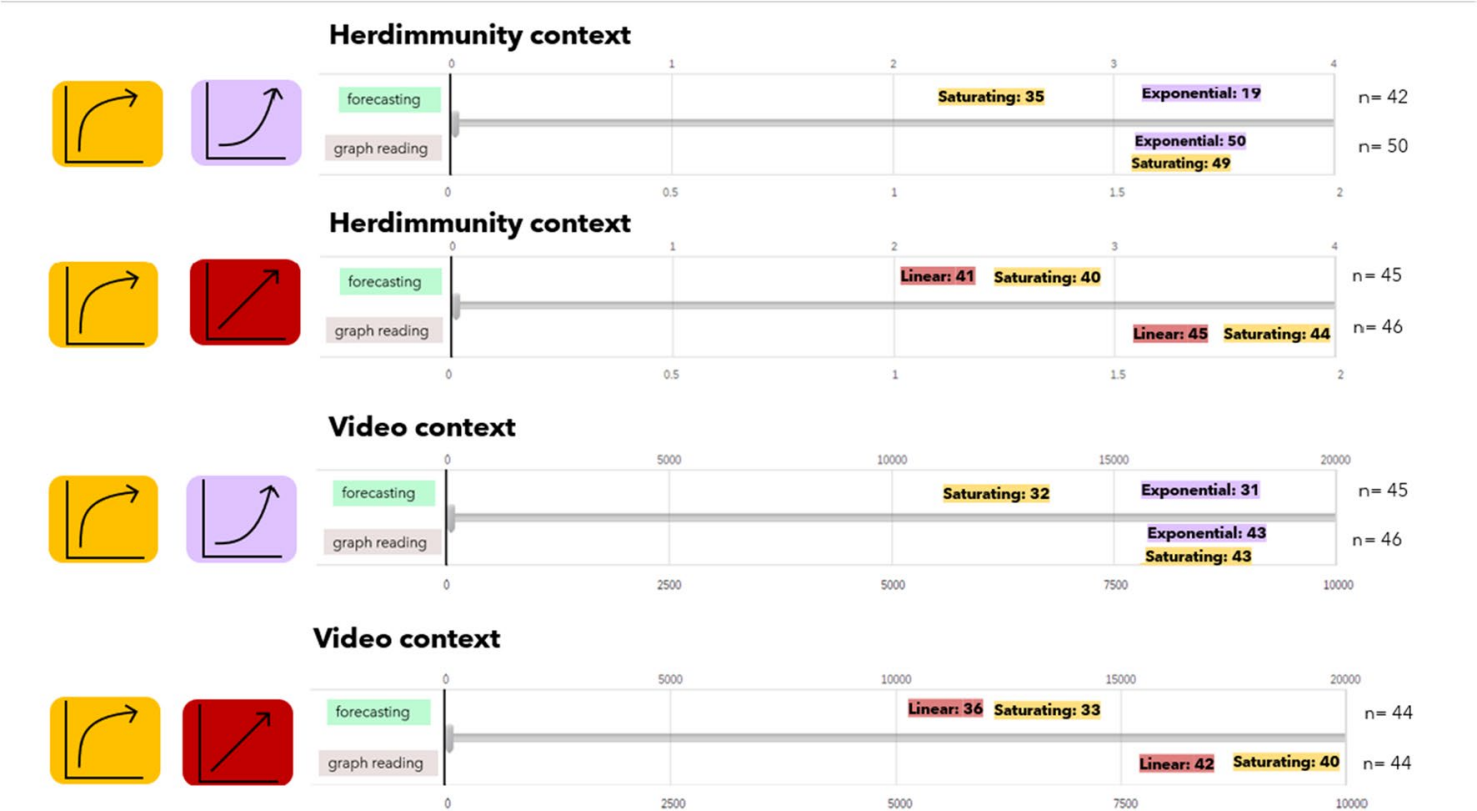
Performance in the forecasting nudge and graph reading active control. In this figure we depict whether the estimates in the two nudge conditions were approximately accurate. The slider scale is split into four quarters and data were coded to see if participants reported a value in the correct quarter for both the forecasting and graph reading conditions. Because the graphs depicted different trends, the correct target value varied across conditions. On the right side of the visualization, we note the number of people viewing each pair of graphs. The number of approximately correct estimations for the exponential, saturating and linear graphs was highlighted with purple, yellow, and terra-cotta color, respectively

In both the graph reading and forecasting nudge conditions, participants reported approximate values via a slider; for each pair of graphs, they reported two estimates depending on the graph type. We wondered whether the relative accuracy of these estimates may have impacted accuracy in the binary decision task. To explore this possibility, we used an unconventional approach to determine whether these estimates were close to target values that needed to be specified in each condition. It was not possible to easily compare error magnitudes across conditions because the target values varied largely across context and graph types. In Fig. 6, we present how many of the estimates fall close to the target range, focusing on whether estimates were in the right quarter. In the next paragraph, we also provide a walk-through for this figure, but as an overview we note that in the graph reading condition, the majority of responses were in the same quarter with the correct value. However, in the forecasting nudge condition, participants were not as successful as graph reading nudge.

Here is a walk-through of Fig. 6: Looking at the first row of this figure, one could see that there were 42 participants in the herd immunity context, who were presented with an exponential-saturating graph pair and were in the forecasting nudge condition. In this group, each participant specified their estimations via a slider separately for exponential graph and saturating graph before choosing one of the two countries as reaching herd immunity sooner (i.e., the binary decision task). While 35 out of 42 participants’ answers were within the same quarter as the correct answer for the saturating graph, only 19 estimations out of 42 were within the same quarter with the correct answer for the exponential graph. Looking at this figure, we see that participants were more accurate in the graph reading task than the forecasting task. Participants were least accurate in the herd immunity context when forecasting and exponential growth and most accurate when forecasting saturating and linear trends in the herd immunity context. This condition is also where we see the greatest impact of the forecasting nudge on binary decision task accuracy (84.4 % vs. 52.4 % for forecast and no-nudge conditions, respectively).

Individual differences such as age, gender, highest education level, majors, etc. were analyzed to see their potential effect on the correct answers rate of binary decision task, but there were no significant effects of any of these variables (see detailed examination in the OSM).

#### Justifications for decisions

Similar to Experiment 1, justifications for decisions in the task were collected with an open-ended question. 480 out of a total of 545 participants provided open-ended answers. Two undergraduate psychology students coded 480 open-ended answers separately into six categories. The inter-rater reliability for these two raters was revealed to be Kappa = .76, p < .001. The raters and HZB resolved conflicts via discussion.

As can be seen in Table 4, the general relationship between choice justifications and accuracy of decisions were similar to Experiment 1. While almost all who referred to the rate of increase were accurate, all of those who focused on early/mid parts of the trajectories were inaccurate in their binary decisions. The only exception was that those who provided linearity-based justifications actually were accurate in Experiment 2. This is likely due to the exclusion of exponential-linear pairs, where focus on linear patterns were likely to mislead viewers. Whether this shows a strong preference for linear trends over others is hard to determine based on these data.

**Table 4:** Open-ended categories according to binary decision task answers

Open-ended categories	False	True	Total
Rate of increase	26 9.9 %	237 90.1 %	263 54.8 %
Focusing on first/middle parts of the graph	66 100 %	0 0%	66 13.8 %
Graph	16 59.3 %	11 40.7%	27 5.6 %
Top-down	57 89.1 %	7 10.9%	64 13.3 %
Linearity	3 6.4 %	44 93.6 %	47 9.8 %
Others	6 46.2 %	7 53.8%	13 2.7%
Total	174 36.3 %	306 63.8 %	480

The table provides the % of accurate responses for each category in the binary decision task. The % values in the far-right column show the % of total answers observed in each category

We inspected the frequency of mention of “rate of increase” category of responses, across nudge and context conditions. Participants referred to the rate of increase on 63.3%, 55.4%, and 46% of the time in the forecasting, graph reading, and no nudge, respectively, χ*2* (10*, N* = 479) = 21.82, *p* = .016, *Cramer’s V* = .151 (see Table 5). When participants referred to “rate of increase” this led to rather high levels of accuracy, regardless of condition 91.6%, 91.4%, and 86.5% for the forecasting, graph reading, and no nudge, respectively. In the no nudge group, 40.8% and 50% of participants reported rate of increase in the herd immunity and video contexts, respectively. In the graph reading nudge, 52.9% and 58.5% of justifications referred to rate of increase. Compared to these two conditions, in the forecasting nudge condition, mention of rate of increase was higher in both contexts: 64.4 % and 62.3 % in herd immunity and video contexts, respectively. Since responses justified by referring to early or mid-parts of the trajectories were always erroneous, we did not inspect these across conditions.

**Table 5:** Justification decisions across nudge conditions

	Decision Justifications						
Nudge	Rate of increase	Focusing on the first and the middle parts of the graph	Graph	Top-down	Linearity	Other	Total
No nudge	74 46.0 %	29 18.0 %	9 5.6 %	30 18.6 %	17 10.6 %	2 1.2 %	161
Graph reading	93 55.4 %	24 14.3 %	9 5.4 %	24 14.3 %	12 7.1 %	6 3.6 %	168
Forecasting	95 63.3 %	13 8.7 %	9 6.0 %	10 6.7 %	18 12.0 %	5 3.3 %	150

#### A closer inspection of the no-nudge condition

**Fig. 7:**
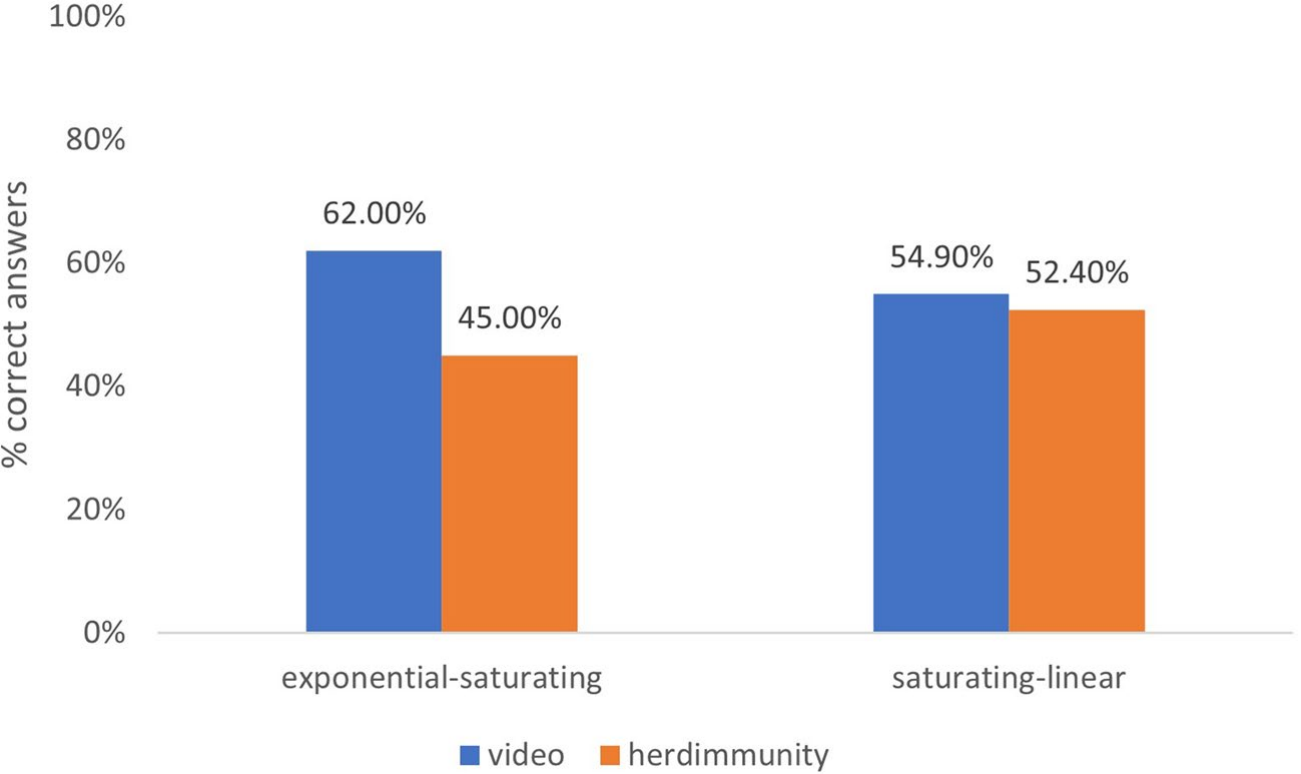
The distributions of Binary Decision Task’s accurate answers across graph pairs for each context in no-nudge condition

The no-nudge condition was procedurally similar to Experiment 1, with one exception – the exclusion of the exponential-linear pair. Therefore, we wanted to determine whether the results mimicked findings from Experiment 1. Chi-square analysis demonstrated that when exponential and saturating graphs were paired, 62% and 45% of participants indicated the correct option in the video and herd immunity contexts, respectively χ^*2*^ (1*, N* = 90) = 2.589, *p* = .11, *Cramer’s V* = .17. For the saturating and linear graph pair, accuracy was similar across the two contexts: video (54.9%) and herd immunity (52.4%), χ^*2*^ (1*, N* = 93) = .059, *p* = .81, *Cramer’s V* = .025 (see Fig. 7). For the exponential-saturating pair, even though we did not find significant differences across two contexts, the direction of the difference (Video > Herd immunity) and magnitude of the effect was similar to Experiment 1 (Cramer’s V = .16 and .17 for Experiment 1 and Experiment 2, respectively). To detect this magnitude of an effect (with .80 power), we would have needed 273 people in this condition; because this was not the primary purpose of Experiment 2, we had not sampled accordingly. We had only 90 participants in the no nudge, exponential-saturating condition. We were not able to replicate the pattern of difference observed for the saturating-linear pair; since our study was not designed and powered as a replication of Experiment 1, we cannot make firm conclusions regarding this particular finding.

## General discussion

Across two experiments, we demonstrated both the challenges associated with processing visually depicted accumulation patterns (Experiment 1) and also effective visual nudges to scaffold accumulation-based reasoning (Experiment 2). In Experiment 1, we demonstrated that understanding accumulation is typically associated with focus on the rate of increase on visual trajectories. We also noted that participants’ misunderstandings of accumulation were driven either by a focus on superficial graph elements or their disregard for the data presented and an overreliance on prior knowledge and beliefs. These biases impacted forecast-based decisions, reducing accuracy. In Experiment 2, we demonstrated that these biases can be eliminated with nudging, as long as the nudge itself was strongly linked to the ultimate decision task. Merely spending time on data reading did not lead to improvements in accumulation-based reasoning.

We report that only the forecasting nudge successfully improved accuracy on the binary decision task; graph reading did not lead to clear advantages. The forecasting nudge might have led participants to think more analytically and critically about the presented data, subsequently improving accuracy on the binary decision task. Analytical thinkers have been shown to be better at understanding accumulation patterns (Hendijani, 2021; Weinhardt et al., 2015). Since the binary decision required making accumulation-based forecasts, the forecasting nudge might have led to improvements in decision accuracy by facilitating an analytic as opposed to superficial and/or schematic processing of the trends presented. The fact that participants were more accurate in the forecasting nudge as opposed to the graph reading nudge, despite both requiring similar amounts of time on task (see OSM) suggests that the improvements are most likely driven by specific nudge characteristics. Merely reaching an accurate answer in the nudge phase did not lead to advantages in the binary decision task. As shown in Fig. 6, participants' estimations in the graph reading nudge were closer to the correct answer. However, the accuracy of estimates did not lead to higher accuracy in the binary decision task. It is possible that the forecasting nudge encouraged participants to “read beyond the data,” whereas the graph reading nudge have only improved “reading the data” (e.g. Friel et al., 2001); the latter might not have been sufficient to help with accumulation-based decisions in the binary task. We should also note that the forecasting nudge shared some similarity to the binary decision task in that both required forecasting accumulation patterns. Since the forecasting nudge may have disrupted intuitive responding during the binary decision task, in this condition participants might have engaged in more effortful cognitive processes compared to participants in the graph reading and no-nudge conditions. In Experiment 1, we had reported a positive relationship between effortful thinking and accuracy. Decision justifications revealed a similar association with accuracy and effortful thinking was only observed in rate of increase category.

Accurate understanding of visual depictions of accumulation patterns and the possible progression of these trends can influence personal decisions and policy support. For instance, accurate understanding of visual depictions of changes in CO2 emissions and absorption and accurately drawn forecasts on these data are critical for increasing support of policies aimed to mitigate climate change (Romano et al., 2020). In the COVID-19 case, understanding visual depictions of total case growth were linked with intentions to continue social distancing (Fansher et al., 2022). In a related vein, understanding risk accumulation in both financial and health contexts and being able to make forecasts based on these data, can lead to better informed decisions (De Bondt, 1993; Sobolev & Harvey, 2016).

We believe that our forecasting nudge that improves understanding of changes in accumulation patterns with time may also be beneficial for stock-flow reasoning. Despite evidence showing that people struggle to understand accumulation and the relationship between stock and flow (Cronin et al., 2009; Dutt & Gonzalez, 2012; Korzilius et al., 2014; Sterman, 2008; Sterman & Sweeney, 2002), research to date has been limited on how to effectively remedy this problem, often with mixed results (e.g., Guy et al., 2013; but also see Newell et al., 2016). In fact, Newell et al. (2013) argued, “Given the low base of accurate performance in stock-flow tasks, any manipulation which leads to over 50% of the sample getting the answer (approximately) correct is newsworthy” (p. 3143). Future work can extend our forecasting nudge idea to stock-flow reasoning paradigms utilizing visual depictions. Since people may not always know where to attend to or how to interpret the data in the visualizations, data walkthroughs and annotations can help them better comprehend how dynamic systems change (e.g., total CO_2_ rate in the atmosphere) (Segel & Heer, 2010; Walls et al., under review). Specifically, it might be possible to improve stock estimates by taking a step-by-step nudging approach to ensure people first understand changes in inflow and outflow patterns, before determining changes in stock.

We believe that future work should test the effectiveness of the forecasting nudge across various contexts. Our choice to use the herd immunity context during an ongoing pandemic might have led people to rely on top-down factors because people were skeptical of the data shared by governments. For instance, subsequent reports revealed that 45% of a nationally representative Turkish sample believed that the data presented by the Ministry of Health and government institutions were not reliable (KONDA, March 2020). If this was the case, participants might have been more tempted to ignore the presented trends in the no-nudge condition and this might have increased the seeming effectiveness of the forecasting nudge. We also believe that future work should investigate the effectiveness of the forecasting nudge when upward trends indicate negative outcomes (e.g., financial loss/debt, death, etc.). There is some work to suggest that forecasts about negative trends are impacted by expectancies of trend-reversal and optimistic tendencies (Harvey & Reimers, 2013). One could think that such biases in forecasts may reduce the effectiveness of the nudge. Even though we believe this is unlikely given our findings from Experiment 2, illustrating that the accuracy on the decision task is not driven by the accuracy of the forecast per se, this remains an open question. Future work can also make use of a broader set of data patterns to ensure the generalizability of findings and use superimposed trajectories to determine whether these graph features may impact forecast-based decision accuracy in the first place.

### Supplementary information

ESM 1 (DOCX 395 kb)

## Footnotes

1 Dynamic systems are characterized by changes in stock as a result of concurrent changes in inflow and outflow (e.g., CO_2_ emissions).

2 We pre-registered the method and anticipated analyses for Experiment 1 on Aspredicted.org, and it can be found at https://aspredicted.org/73G_QC2 . See the notes in the OSM for deviations from our pre-registrations.

3 We also conducted Logistic Regression Analyses and they are available in the OSM. The general pattern of findings is similar. See OSM for details.
