# Supplementary material for: Understanding patterns of accumulation: Improving forecast-based decisions via nudging
Source: Mem Cognit. 2024 Jan 25;52(5):1033–47. doi: 10.3758/s13421-024-01519-6 (PMC11315707; doi:10.3758/s13421-024-01519-6)
Supplement: Supplementary file 1 — (DOCX 395 kb) [file 13421_2024_1519_MOESM1_ESM.docx]

**Supplemental Information**

This document includes:

**Notes i to iv**

**Supplemental Methods**

Graph reading and forecasting nudge displays

**Supplementary Results**

Descriptives of participants’ total durations during the study in Experiment 1

Detailed information about the sample in Experiment 1

The relationship between binary decision task and graph understanding task

Detailed examination of individual differences for the binary decision task in Experiment 1

Descriptives of participants’ total durations during the study in Experiment 2

Detailed information about the sample in Experiment 2

Durations comparison across nudge conditions in Experiment 2

Accuracy in the binary decision task for different contexts and graph pairs in Experiment 2

The distributions of binary decision task’s accurate answers across nudge conditions for each context in Experiment 2

Detailed examination of individual differences for the binary decision task in Experiment 2

Decision justification categories and main keywords

Logistic Regression Analysis Results

**All the R formulas used to create displays in Experiment 1 and Experiment 2**

**SPSS syntax for Experiment 1 and Experiment 2**

**OSF link for datasets**

**Notes**

^i^ In several ways, we deviated from the preregistrations we made for Experiment 1. In the preregistration, we had stated that accurate decisions would increase when viewing the exponentially increasing graph with a saturated graph compared to a linear graph. Also, we mentioned that accurate decisions would increase when viewing the saturated graph with a linearly increasing graph compared to exponentially increasing graph. However, there seemed very scant literature to justify these predictions. In the current version of the manuscript, instead of comparing the accuracies of different pair groups, we decided to look at the accuracy level for each pair. Therefore, we present nonparametric contingency tables (Chi-Square).

^ii^ Although we preregistered hypotheses related to Comprehensive Thinking Scale and graph understanding task in Experiment 1, they were not the primary focus of the study, thus the findings of associated analyses were included in the Supplemental Materials.

^iii^ One of the exclusion criteria mentioned in the preregistration report to identify outliers was if the Comprehensive Thinking Scale score was extreme (more than 3 standard deviations), we would exclude them from further analyses, specifically for those analyses involving thinking style. However, we did not employ this preregistered criterion, because this variable was not the primary focus of the study and all relevant analyses were included in the supplemental materials. Another preregistered exclusion criterion was that we would check the time spent reading the scenarios and exclude any participant who read the scenario explanations in a very short amount of time. Instead of doing this, we examined how much time participants spent on the entire experiment and used the trimming procedure to exclude those who spent either too much or too little time.

^iv^ There is also deviation for the preregistered sample size statement. We thought of collecting data from 300 participants with the intention of using logistic regression analysis. However, we made the decision to leave the Qualtrics experiment link accessible for a longer amount of time when we started to get incomplete experiment data during the online data collection phase. As a result, we completed the data collecting procedure with 1081 clicks (only 710 of participants completed the main tasks).

**Supplemental Methods**

***Graph reading and forecasting nudge displays***

**Figure S1**

*Graph reading nudge displays*

*
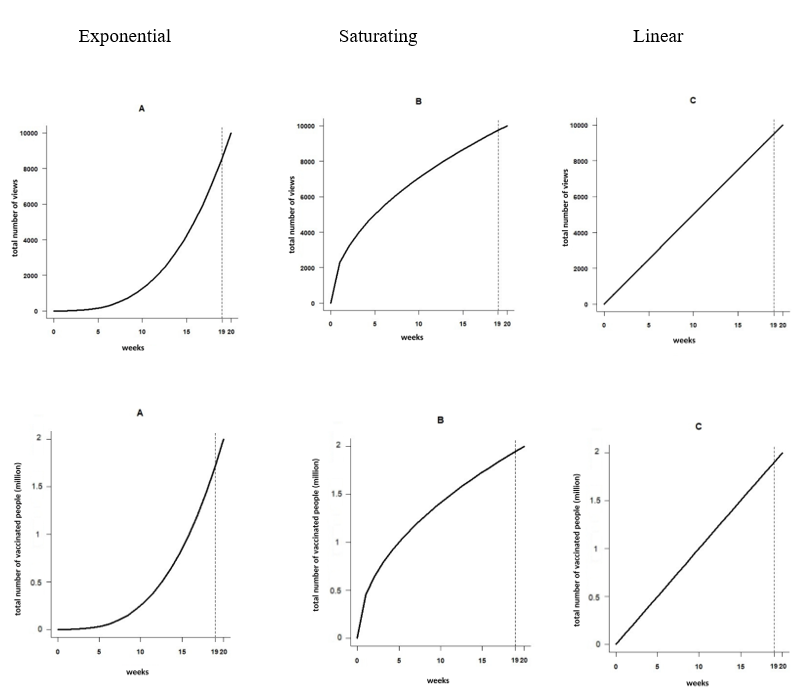
*

*Note.* The top panel show the graphs used in video context while the bottom panel show the graphs shown in the herd immunity context. In all graphs, the x-axis depicted weeks; the y-axis depicted the total number of video viewings or total number of vaccinated individuals, respectively. The y axis numbers on the graphs in the herd immunity condition are displayed in millions.

**Figure S2**

*Forecasting nudge displays*
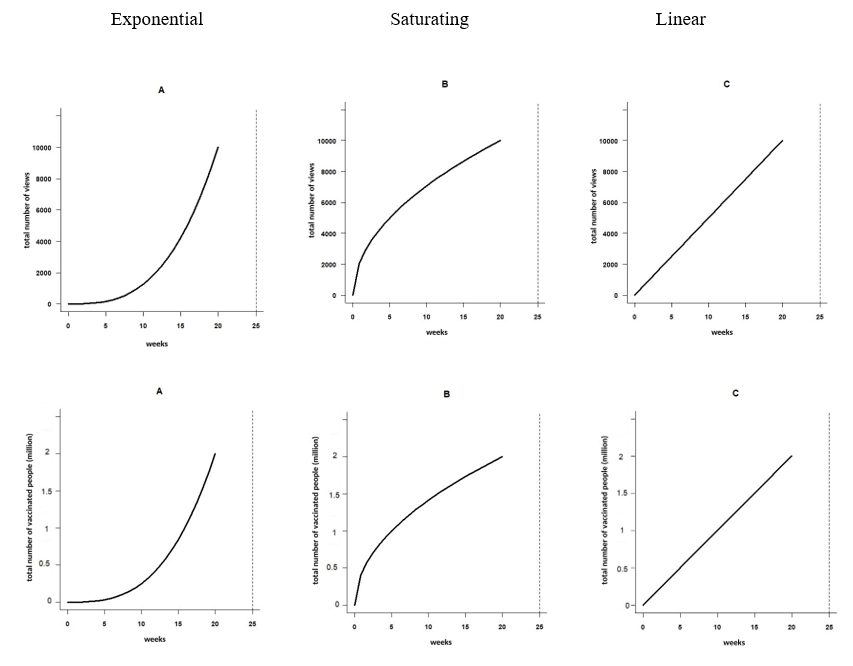
*Note.* The top panel show the graphs used in video context while the bottom panel show the graphs shown in the herd immunity context. In all graphs, the x-axis depicted weeks; the y-axis depicted the total number of video viewings or total number of vaccinated individuals, respectively. The y axis numbers on the graphs in the herd immunity condition are displayed in millions.

**Supplementary Results**

***Descriptives of participants’ total durations during the study in Experiment 1***

**Table S1**

| *Descriptives of duration (in seconds) in Experiment 1 before 5% trimming* | | | |
| --- | --- | --- | --- |
|  |  |  |  |
|  | | **Duration (in seconds)** | |
| N |  | 710 |  |
| Missing |  | 0 |  |
| Mean |  | 1207.89 |  |
| 5% Trimmed Mean  Median |  | 401.03  366.5 |  |
| Standard deviation |  | 6956.49 |  |
| Variance |  | 48392751.068 |  |
| IQR |  | 205.5 |  |
| Range |  | 93678 |  |
| Minimum |  | 106 |  |
| Maximum |  | 93784 |  |
| Skewness |  | 10.4 |  |
| Std. error skewness |  | 0.0917 |  |
| Kurtosis |  | 113 |  |
| Std. error kurtosis |  | 0.183 |  |
|  | | | |

***Detailed information about the sample in Experiment 1***

The majority of the participants in the sample were undergraduate and graduate students (Table S2). Distributions of students participated in the Experiment 1 are respectively, 43.4% from quantitative areas, 36.7% from equally weighted areas, and 18.7% from qualitative or language-related ones. Most of the participants held either a bachelor's degree or a master’s degree/PhD (Table S2). In addition, student participants indicated the number of mathematics courses taken at university (from “0” to “4 or more”). Almost half of the student participants (45.9%) did not take any mathematics courses at university.

| **Table S2**  *Frequencies of undergraduates, graduates, and non-students* | | | |
| --- | --- | --- | --- |
| **Levels** | **Counts** | **% of Total** | **Cumulative %** |
| Non-student – high school degree or lower | 17 | 2.7 % | 2.3 % |
| Non-student – college degree or higher | 189 | 29.5 % | 32.3% |
| Undergraduate student | 288 | 45 % | 77.4 % |
| Graduate Student | 144 | 22.5 % | 100.0 % |

***The relationship between binary decision task and graph understanding task***

Participants were asked about the last week in the second task: which video or country had the highest viewing or vaccinated people rate in the last week. As an expletory analysis to examine whether there is a significant relationship between the answers of the binary decision task and graph understanding task, we conducted correlation analysis. There was a weak and non-significant correlation between the answers of the binary decision task and graph understanding task questions, *phi* =.068, *p* = .083.

***Detailed examination of individual differences for the binary decision task in Experiment 1***

Accuracy rates of the binary decision task were affected by the highest education level of participants; high school (65.6%), undergraduate (58%) or graduate students (75%), *ꭓ^2^* (2*, N* = 634) = .6.142, *p* = .046, *Cramer’s V* = .098. However, when we divided student participants into two groups via median split, as the group which did not take any mathematics courses and which did take mathematics courses, the effect of taken mathematics courses on the accuracy rates could not be observed, *ꭓ^2^* (1*, N* = 408) = .232, *p* = .63, *Cramer’s V* = .024.

In addition, departments of the student participants significantly affected the accuracy rates in the task 1, *ꭓ^2^* (2,*N* = 413) = 9.348, *p* = .009, *Cramer’s V* = .150. Correct answer percentage given by the students from three different group of majors are respectively, 54.4% for qualitative major students, 59.7% for quantitative major students, and 72.5% for equally weighted major students.

We also investigated whether the gender and age variables affect accuracy rates in the tasks. While 58.8% of the female participants gave correct answers, 73.9% of the male participants responded with accurate answers in the task 1, *ꭓ^2^* (1*, N* = 631) = 10.42, *p* = .001, *Cramer’s V* = .129.When the gender effect was examined in a detailed way, the difference of accurate answers was not observed in the group of participants whose last education level either college or master’s degree/PhD, (all *p*s < .05) but 81% of the male participants whose highest degree is high school gave the correct answer to the binary decision task question whereas 61.1% of the female participants whose highest degree is high school indicated correct answers, *ꭓ^2^* (1*, N* = 302) = 8.675, *p* = .003, *Cramer’s V* = .169. This gender difference between male and female groups on the task accuracy may indeed stem from the female sample containing more participants whose majors were related to qualitative or language areas. While only 10.7% of the male participants studied qualitative majors, 21.5% of female participants’ majors were qualitative related areas. Lastly, A point-biserial correlation was run to see the relationship between age and the answers of the binary decision task. No significant relationship was observed between the age and task accuracy of participants, *r_pb_* = .015, *n* = 640, *p* = .711

***Descriptives of participants’ total durations during the study in Experiment 2***

**Table S3**

| *Descriptives of duration (in seconds) in Experiment 2 before 5% trimming* | | | |
| --- | --- | --- | --- |
|  |  |  |  |
|  | | **Duration (in seconds)** | |
| N |  | 605 |  |
| Missing |  | 0 |  |
| Mean |  | 1099.55 |  |
| 5% Trimmed Mean  Median |  | 352.97  293 |  |
| Standard deviation |  | 5462.2 |  |
| Variance |  | 29835639.2 |  |
| IQR |  | 225 |  |
| Range |  | 78795 |  |
| Minimum |  | 30 |  |
| Maximum |  | 78825 |  |
| Skewness |  | 9.67 |  |
| Std. error skewness |  | 0.099 |  |
| Kurtosis |  | 105.81 |  |
| Std. error kurtosis |  | 0.198 |  |

***Detailed information about the sample in Experiment 2***

Undergraduate and graduate students constituted the majority of the participants (Table S4). 37.7% of students’ majors were quantitative areas, 43% of them were from equally weighted areas, and 19.3% were from qualitative or language-related ones. When we examined the non-student sample in a detailed way, it was observed that most of the participants had either a bachelor's degree or a master’s degree/PhD (90.2%). In addition, participants indicated the number of mathematic courses taken at university (from “0” to “4 or more”). Almost half of the students (52.6%) in the study either did not take any mathematics courses in university or took only one.

| **Table S4**  *Frequencies of undergraduates, graduates, and non-students* | | | |
| --- | --- | --- | --- |
| **Levels** | **Counts** | **% of Total** | **Cumulative %** |
| Non-student – high school degree or lower | 21 | 4.13% | 4.13% |
| Non-student – college degree or higher | 193 | 38.1% | 42.2% |
| Undergraduate student | 189 | 37.2 % | 79.5 % |
| Graduate Student | 104 | 20.05 % | 100.0 % |

|  |
| --- |

***Durations comparison across nudge conditions in Experiment 2***

One way between subject ANOVA indicated that total durations of participants were significantly different, *F* (2, 542) = 12.67, *p* < .001. Post-hoc comparisons using Tukey HSD test revealed that while participants in no nudge group (*M* = 290.79, *SD* = 14.46) spent less time compared to both participants in forecasting (*M* = 372.37, *SD* = 15.31), and graph reading nudge (*M* = 397.34, *SD* = 17.19), conditions whereas durations or forecasting and graph reading conditions did not differ from each other (p values for post-hoc comparisons respectively, *p* = .001, *p* < .001, *p* = .50)

***Accuracy in the binary decision task for different contexts and graph pairs in Experiment 2***

**Table S5**

*The results of chi-square analyses for the context and graph pair comparisons*

|  | Context | Graph Types | *Accuracy rate* | *DF* | *Chi-square value* | | | *p-value* | *Cramer’s V* | | |
| --- | --- | --- | --- | --- | --- | --- | --- | --- | --- | --- | --- |
| Context | Video vs Herd immunity | ------------ | 63.2% - 60.4% | 1, 545 | .464 | | | .50 | .029 | | |
|  |  |  |  |  |  | | |  |  | | |
| Graph pair | ----------- | Exponential- Saturating vs Saturating-Linear | 61.5% - 62.1% | 1, 545 | .20 | | | .89 | .006 | | |
|  |  |  |  |  |  | | |  |  | | |
| Context* Graph pair | Video vs Herd immunity | Exponential- Saturating | 66% - 56.8% | 1, 273 | 2.406 | | | .11 | .094 | | |
| Context* Graph pair | Video vs Herd immunity | Saturating- Linear | 60.4% - 63.9% | 1, 348 | .349 | | | .54 | .036 | | |
|  |  |  |  |  | |  |  | | |  |  |

*Note.* For the context and graph pair comparison we collapsed a cross graph type and context respectively (indicated by the dotted lines). For the cross tabulation of the context and graph pair, top row looked at the impact for exponential-saturating graph pair only and bottom row saturating-linear pair.

***The distributions of Binary Decision Task’s accurate answers across nudge conditions for each context in Experiment 2***

Chi-square analyses were conducted for each graph pair condition across contexts to investigate the influence of nudges on accuracy. In the herd immunity context, among the participants who viewed saturating and linear graph pair, the ones took forecasting nudge responded with a higher rate of correct answers compared to graph reading and no nudge condition, *ꭓ^2^* (2*, N* = 133) = 12.471, *p* = .002, *Cramer’s V* = .31. Despite the similar trend was observed for the exponential and saturating pair group, the chi-square analysis result was not significant, *ꭓ^2^* (2*, N* = 132) = 3.966, *p* = .138, *Cramer’s V* = .173. On the other hand, when the effect of nudges were examined in a video context for each graph pair condition, neither in the exponential-saturating graph pair (*ꭓ^2^* (2*, N* = 141) = 2.725, *p* = .256, *Cramer’s V* = .139) nor in the saturating-linear graph pair (*ꭓ^2^* (2*, N* = 139) = 1.791, *p* = .409, *Cramer’s V* = .113) significant differences observed. However, despite the insignificant results, as the Figure S3 clearly shows that participants who were in the forecasting nudge condition responded with a higher rate of correct answers in the binary decision task compared to the other two conditions.

**Figure S3**

*The distributions of Binary Decision Task’s accurate answers across nudge conditions for each context*

***Detailed examination of individual differences for the binary decision task in Experiment 2***

When we divided student participants into two groups via median split as the group which did not take any mathematics courses or only took one course, and who took 2, 3, or more mathematics courses, the effect of taken mathematics courses on accuracy rates could not be observed, *ꭓ^2^* (1*, N* = 477) = 1.57, *p* = .21, *Cramer’s V* = .057. In addition, the majors of the student participants did not significantly affect the accuracy rates in the task, *ꭓ^2^* (2*, N* = 477) = 4.202, *p* = .112, *Cramer’s V* = .094. Correct answer percentage given by the students from three different group of majors are respectively, 55.4% for qualitative major students, 67.8% for quantitative major students, and 65.4% for equally weighted major students. Lastly, accuracy rates of the binary decision task were not affected by the highest education level of participants or by participants being a student or non-student, (all *p*s > .05)

We also investigated whether the gender and age variables affect accuracy rates in the task. While 62.3% of the female participants gave correct answers, 66.4% of the male participants indicated accurate answers in the task, *p* > .05. Lastly, A point-biserial correlation was run to see the relationship between age and the answers of the binary decision task. There were no significant relationships between age and task accuracy, *p* > .05.

***Decision justification categories and main keywords***

**Table S6**

*Decision justification categories and main keywords*

| **Coding number** | **Category** | **Main Keywords** |
| --- | --- | --- |
| 1 | Rate of increase | slope, plateau, recent increase, rate of increase, decreasing increase, exponential increase |
| 2 | Focusing on the first or the middle parts of the graph | curve is higher, looking at the first weeks, looking at the middle |
| 3 | Graph | mentioned just looking at the graph |
| 4 | Relying on top-down | vaccine supply, knowledge about vaccines, being viral,the nature of advertising  etc. |
| 5 | Linearity | linearity, stability, cautious growth, regularity |
| 6 | Other | - |

A single category was employed to code each open-ended response in the decision justification. Some answers might fit in more than one category. Based on the response's main theme, we tried to place such instances in the most relevant category. There were some justifications, yet, where it was unclear which category predominated; in these cases, we used the following strategy: Each category has specific keywords associated with it. In cases where a justification could be assigned to multiple categories, we prioritized the category based on the occurrence of the keywords specifically related to that category in the justification. See the main keywords above.

***Logistic Regression Analysis Results***

Early on in our data analyses, we considered to use logistic regression analysis, but we decided against this approach. When we included the key variables into the models, our inspection of the ROC curves (the sensitivity vs. specificity graphs) revealed that the logistic regression solutions did not offer good enough fits to discriminate accurate from inaccurate responses in the binary decision task. However, we provide the results of this analysis for both Experiment 1 and Experiment 2 by highlighting ROC showing the models’ weak predictive power.

A logistic regression analysis for Experiment 1 was conducted to examine the effects of graph pair types, context, effortful thinking, and the interaction between context and graph pair types on the likelihood of accuracy in the binary decision task. The model was statistically significant, *χ²(*6, *N* = 640) = 20.5, *p* = .002. However, it explained very modest proportion of the variance in task accuracy, Nagelkerke *R*² = 3.87%. Graph pair types did not significantly predict accuracy (*p* = .309), nor did effortful thinking (*p* = .134). However, context was shown to be a significant predictor (*p* = .002), and there was a notable interaction between context and graph pair types (*p* = .001), indicating that the influence of graph pair type on accuracy varies by context.

There was a lower likelihood of accurate task performance when in the video context (OR = 0.442, 95% CI [0.260, 0.752], *p* = .003). The results of the interactions showed that this effect was more prominent for certain types of graph pairs: the odds of accurate decisions in the video context for the exponential-saturating vs. saturating-linear graph pair was 3.892, 95% CI [1.840, 8.232], *p* < .001, and for the exponential-linear vs. saturating-linear graph pair was 2.442, 95% CI [1.137, 5.246], *p* = .022.

The examination of the ROC curve revealed an AUC of 0.595, which, while above the chance level, suggests that the model's predictive power is restricted (See Figure S4).

**Figure S4**

*ROC Curve for Experiment 1*


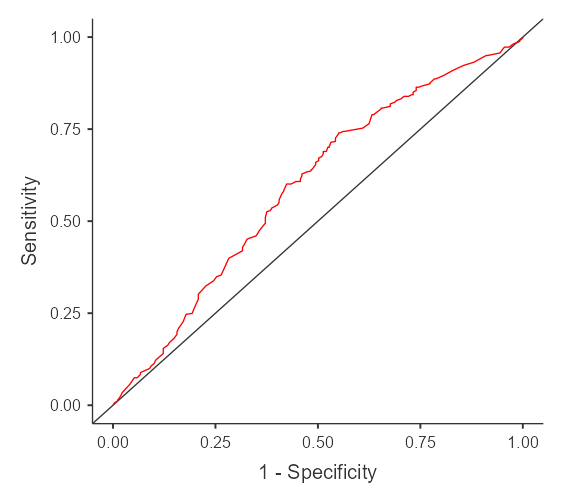


A logistic regression analysis for Experiment 2 was conducted to examine the effects of nudge, graph pair and context on the likelihood of participants to make accurate decisions in a binary decision task. First of all, the model was statistically significant, *χ*²(4, *N* = 545) = 17.7, *p* = .001. The model explained 4.3% (Nagelkerke *R*²) of the variance in decision accuracy.

Participants in the forecasting nudge condition were more likely to make accurate decisions than those in the no-nudge condition (OR=2.42, 95% CI [1.548, 3.77]). Decision accuracy was not significantly predicted by the kind of graph pair or the context, *p* = .898 and *p* = .433, respectively. The probability of making an accurate decision was 53.9% in the no-nudge condition, 58.1% in the graph reading nudge condition, and 73.9% in the forecasting nudge condition.

The model’s prediction accuracy is only slightly better than chance as evidenced by the area under the ROC curve (AUC) of .60 (See Figure S5). The marginal differentiation implies that the model’s ability to distinguish between correct and incorrect decisions is not very strong.

**Figure S5**

*ROC Curve for Experiment 2*


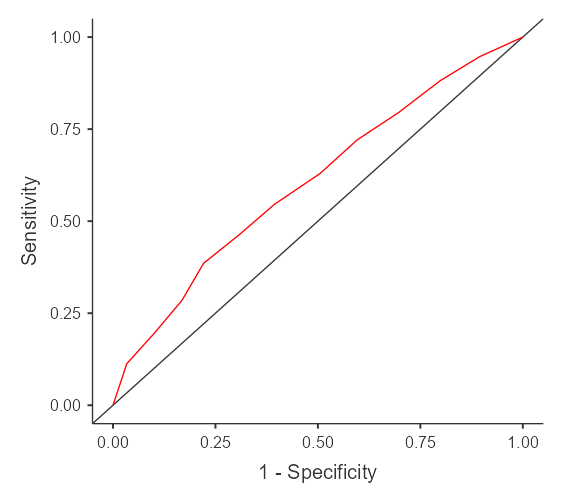


***R formulas used in Experiment 1 to create the displays***

**#herd immunity context**

op <- par(mfrow = c(1, 2))

options(scipen = 100)

curve( 250*x^3, from=0, to=20, n=20, font = 2, main = "A", xlab="weeks", ylab="total number of vaccinated people",

col="black", lwd=3, cex.axis = 1, cex.lab = 1.1)

curve( 447213.5955*x^0.5, from=0, to=20, n=20, font = 2, main = "B", xlab="weeks", ylab="total number of vaccinated people",

col="black", lwd=3, cex.axis = 1, cex.lab = 1.1)

curve( 100000*x, from=0, to=20, n=20, font = 2,, main = "C", xlab="weeks", ylab="total number of vaccinated people",

col="black", lwd=3, cex.axis = 1, cex.lab = 1.1)

**#video context**

op <- par(mfrow = c(1, 2))

options(scipen = 100)

curve( 1.25*x^3, from=0, to=20, n=20, font = 2, main = "A", xlab="weeks", ylab="total number of views",

col="black", lwd=3, cex.axis = 1, cex.lab = 1.1)

curve( 2236.0679775*x^0.5, from=0, to=20, n=20, font = 2, main = "B", xlab="weeks", ylab="total number of views",

col="black", lwd=3, cex.axis = 1, cex.lab = 1.1)

curve( 500*x, from=0, to=20, n=20, font = 2, main = "C", xlab="weeks", ylab="total number of views",

col="black", lwd=3, cex.axis = 1, cex.lab = 1.1)

***R formulas used in Experiment 2 to create the displays***

**####forecasting nudge####**

**#herd immunity context**

op <- par(mfrow = c(1, 2), bty = "L", main = "bty = 'L'")

options(scipen = 100)

curve( 250*x^3, from=0, to=20, n=25, font = 2, main = "A", xlab="weeks", ylab="total number of vaccinated people (million)",

col="black", lwd=3, cex.axis = 0.9, cex.lab = 1.1, xlim = c(0,25), ylim=range(0:2500000), xaxt="s")

abline(v=25, col="black", type="l", lty=2)

curve( 447213.5955*x^0.5, from=0, to=20, n=25, font = 2, main = "B", xlab="weeks", ylab="total number of vaccinated people (million)",

col="black", lwd=3, cex.axis = 0.9, cex.lab = 1.1, xlim = c(0,25),ylim=range(0:2500000), xaxt="s")

abline(v=25, col="black", type="l", lty=2)

curve( 100000*x, from=0, to=20, n=25, font = 2,, main = "C", xlab="weeks", ylab="total number of vaccinated people (million)",

col="black", lwd=3, cex.axis = 0.9, cex.lab = 1.1, xlim = c(0,25), ylim=range(0:2500000), xaxt="s")

abline(v=25, col="black", type="l", lty=2)

#In the 25th week, the exponential graph will arrive at this value: 250*25^3=3906250

#In the 25th week, the saturating graph will arrive at this value.:447213.5955*25^0.5= 2236067.9775

#In the 25th week, the linear graph will arrive at this value.: 100000*25=2500000

**#video context**

op <- par(mfrow = c(1, 2), bty = "L", main = "bty = 'L'")

options(scipen = 100)

curve( 1.25*x^3, from=0, to=20, n=25, font = 2, main = "A", xlab="weeks", ylab="total number of views",mgp=c(3.3,1,0),

col="black", lwd=3, cex.axis = 0.9, cex.lab = 1.1, xlim = c(0,25),ylim=range(0:12000), las = 1, xaxt="s")

abline(v=25, col="black", type="l", lty=2)

curve( 2236.0679775*x^0.5, from=0, to=20, n=25, font = 2, main = "B", xlab=" weeks", ylab="total number of views",mgp=c(3.3,1,0),

col="black", lwd=3, cex.axis = 0.9, cex.lab = 1.1, xlim = c(0,25), ylim=range(0:12000), las = 1, xaxt="s")

abline(v=25, col="black", type="l", lty=2)

curve( 500*x, from=0, to=20, n=25, font = 2, main = "C", xlab=" weeks", ylab="total number of views",mgp=c(3.3,1,0),

col="black", lwd=3, cex.axis = 0.9, cex.lab = 1.1, xlim = c(0,25), ylim=range(0:12000), las = 1, xaxt="s")

abline(v=25, col="black", type="l", lty=2)

#In the 25th week, the exponential graph will arrive at this value: 1.25 x 25^3=19531.25

#In the 25th week, the saturating graph will arrive at this value: 2236.0679775*25^0.5=11180.3398875

#In the 25th week, the exponential graph will arrive at this value 500*25= 12500

**####graph reading nudge####**

**#herd immunity context**

op <- par(mfrow = c(1, 2))

options(scipen = 100)

curve( 250*x^3, from=0, to=20, n=20, font = 2, main = "A", xlab="weeks", ylab="total number of vaccinated people (million)",

col="black", lwd=3, cex.axis = 0.9, cex.lab = 1.1, xaxt="s")

abline(v=19, col="black", type="l", lty=2)

curve( 447213.5955*x^0.5, from=0, to=20, n=20, font = 2, main = "B", xlab=" weeks", ylab="total number of vaccinated people (million)",

col="black", lwd=3, cex.axis = 0.9, cex.lab = 1.1, xaxt="s")

abline(v=19, col="black", type="l", lty=2)

curve( 100000*x, from=0, to=20, n=20, font = 2,, main = "C", xlab=" weeks", ylab="total number of vaccinated people (million)",

col="black", lwd=3, cex.axis = 0.9, cex.lab = 1.1, xaxt="s")

abline(v=19, col="black", type="l", lty=2)

**#video context**

op <- par(mfrow = c(1, 2))

options(scipen = 100)

curve( 1.25*x^3, from=0, to=20, n=20, font = 2, main = "A", xlab="weeks", ylab="total number of views", mgp=c(3.3,1,0),

col="black", lwd=3, cex.axis = 0.9, cex.lab = 1.1,las = 1, xaxt="s")

abline(v=19, col="black", type="l", lty=2)

curve( 2236.0679775*x^0.5, from=0, to=20, n=20, font = 2, main = "B", xlab="weeks", ylab="total number of views", mgp=c(3.3,1,0),

col="black", lwd=3, cex.axis = 0.9, cex.lab = 1.1, las = 1,xaxt="s")

abline(v=19, col="black", type="l", lty=2)

axis(1, at=1,labels=19

curve( 500*x, from=0, to=20, n=20, font = 2,, main = "C", xlab=" weeks", ylab="total number of views", mgp=c(3.3,1,0),

col="black", lwd=3, cex.axis = 0.9, cex.lab = 1.1, las = 1, xaxt="s")

abline(v=19, col="black", type="l", lty=2)

***Experiment 1 SPSS codes***

“task1_accuracy” variable refers to the binary decision task accuracy in Experiment 1 and was coded as true or false.

“task2_accuracy” variable refers to the graph understanding task accuracy in Experiment 1 and was coded as true or false. (These analysis results were included in Supplementary document)

“context” variable was coded as video or herd immunity while “graph_pairs” variable was coded as AB (exponential vs saturating), AC (exponential vs linear) or BC (saturating vs linear).

FREQUENCIES VARIABLES=gender age student_or_not lastlevel_education majors_coded

math_course

/STATISTICS=STDDEV VARIANCE MINIMUM MAXIMUM MEAN MEDIAN MODE

/ORDER=ANALYSIS.

CROSSTABS

/TABLES=context BY task1_accuracy

/FORMAT=AVALUE TABLES

/STATISTICS=CHISQ PHI CORR

/CELLS=COUNT ROW

/COUNT ROUND CELL.

CROSSTABS

/TABLES=graph_pairs BY task1_accuracy

/FORMAT=AVALUE TABLES

/STATISTICS=CHISQ PHI CORR

/CELLS=COUNT ROW

/COUNT ROUND CELL.

CROSSTABS

/TABLES=context BY task1_accuracy BY graph_pairs

/FORMAT=AVALUE TABLES

/STATISTICS=CHISQ PHI CORR

/CELLS=COUNT ROW

/COUNT ROUND CELL

/BARCHART.

CROSSTABS

/TABLES=graph_pairs BY task1_accuracy BY context

/FORMAT=AVALUE TABLES

/STATISTICS=CHISQ PHI CORR

/CELLS=COUNT ROW

/COUNT ROUND CELL

/BARCHART.

CROSSTABS

/TABLES=coder1 BY coder2

/FORMAT=AVALUE TABLES

/STATISTICS=CHISQ PHI KAPPA

/CELLS=COUNT

/COUNT ROUND CELL.

CROSSTABS

/TABLES=open_ended_coding_final BY task1_accuracy

/FORMAT=AVALUE TABLES

/STATISTICS=CHISQ CC PHI

/CELLS=COUNT ROW COLUMN

/COUNT ROUND CELL.

CROSSTABS

/TABLES=task1_accuracy BY task2_accuracy

/FORMAT=AVALUE TABLES

/STATISTICS=CHISQ PHI LAMBDA CORR

/CELLS=COUNT

/COUNT ROUND CELL.

EXAMINE VARIABLES=majors_coded BY gender

/PLOT BOXPLOT STEMLEAF

/COMPARE GROUPS

/STATISTICS DESCRIPTIVES

/CINTERVAL 95

/MISSING LISTWISE

/NOTOTAL.

EXAMINE VARIABLES=majors_coded

/PLOT BOXPLOT STEMLEAF

/COMPARE GROUPS

/STATISTICS DESCRIPTIVES

/CINTERVAL 95

/MISSING LISTWISE

/NOTOTAL.

CROSSTABS

/TABLES=gender BY task1_accuracy

/FORMAT=AVALUE TABLES

/STATISTICS=CHISQ PHI CORR

/CELLS=COUNT ROW TOTAL

/COUNT ROUND CELL.

CROSSTABS

/TABLES=number_of_math BY task1_accuracy

/FORMAT=AVALUE TABLES

/STATISTICS=CHISQ PHI CORR

/CELLS=COUNT ROW

/COUNT ROUND CELL.

NONPAR CORR

/VARIABLES=task1_accuracy intuitive_thinking effortful_thinking

/PRINT=SPEARMAN TWOTAIL NOSIG

/MISSING=PAIRWISE.

***Experiment 2 SPSS codes***

“accuracy” variable refers to the binary decision task accuracy in Experiment 2 and was coded as true or false.

“context” variable was coded as video or herd immunity while “graph_pair” variable was coded as AB (exponential vs saturating) or BC (saturating vs linear).

“nudge” variable was coded as no nudge, graph reading or forecasting.

DESCRIPTIVES VARIABLES=age

/STATISTICS=MEAN STDDEV VARIANCE RANGE MIN MAX KURTOSIS SKEWNESS.

FREQUENCIES VARIABLES=gender

/ORDER=ANALYSIS.

FREQUENCIES VARIABLES=participated_Exp1 student_or_not lastlevel_education majors_coded

/ORDER=ANALYSIS.

FREQUENCIES VARIABLES=Math_courses

/ORDER=ANALYSIS.

CROSSTABS

/TABLES=nudge BY accuracy

/FORMAT=AVALUE TABLES

/STATISTICS=CHISQ PHI

/CELLS=COUNT ROW

/COUNT ROUND CELL

/BARCHART.

CROSSTABS

/TABLES=nudge BY accuracy BY context

/FORMAT=AVALUE TABLES

/STATISTICS=CHISQ PHI

/CELLS=COUNT ROW

/COUNT ROUND CELL

/BARCHART.

CROSSTABS

/TABLES=nudge BY accuracy BY graph_pair BY context

/FORMAT=AVALUE TABLES

/STATISTICS=CHISQ PHI

/CELLS=COUNT ROW

/COUNT ROUND CELL

/BARCHART.

ONEWAY Duration__in_seconds_ BY nudge

/STATISTICS DESCRIPTIVES HOMOGENEITY WELCH

/MISSING ANALYSIS

/POSTHOC=TUKEY ALPHA(0.05).

EXAMINE VARIABLES=Duration__in_seconds_ BY nudge

/PLOT BOXPLOT HISTOGRAM

/COMPARE GROUPS

/STATISTICS DESCRIPTIVES

/CINTERVAL 95

/MISSING LISTWISE

/NOTOTAL.

CROSSTABS

/TABLES=context BY accuracy

/FORMAT=AVALUE TABLES

/STATISTICS=CHISQ PHI

/CELLS=COUNT ROW

/COUNT ROUND CELL

/BARCHART.

CROSSTABS

/TABLES=graph_pair BY accuracy

/FORMAT=AVALUE TABLES

/STATISTICS=CHISQ PHI

/CELLS=COUNT ROW

/COUNT ROUND CELL

/BARCHART.

CROSSTABS

/TABLES=context BY accuracy BY graph_pair

/FORMAT=AVALUE TABLES

/STATISTICS=CHISQ PHI

/CELLS=COUNT ROW

/COUNT ROUND CELL

/BARCHART.

USE ALL.

COMPUTE filter_$=(nudge = 1 | nudge = 2).

VARIABLE LABELS filter_$ 'nudge = 1 | nudge = 2 (FILTER)'.

VALUE LABELS filter_$ 0 'Not Selected' 1 'Selected'.

FORMATS filter_$ (f1.0).

FILTER BY filter_$.

EXECUTE.

CROSSTABS

/TABLES=nudge BY accuracy BY context

/FORMAT=AVALUE TABLES

/STATISTICS=CHISQ CC PHI ETA

/CELLS=COUNT ROW

/COUNT ROUND CELL.

USE ALL.

COMPUTE filter_$=(nudge = 1 | nudge = 3).

VARIABLE LABELS filter_$ 'nudge = 1 | nudge = 3 (FILTER)'.

VALUE LABELS filter_$ 0 'Not Selected' 1 'Selected'.

FORMATS filter_$ (f1.0).

FILTER BY filter_$.

EXECUTE.

CROSSTABS

/TABLES=nudge BY accuracy BY context

/FORMAT=AVALUE TABLES

/STATISTICS=CHISQ CC PHI ETA

/CELLS=COUNT ROW

/COUNT ROUND CELL.

USE ALL.

COMPUTE filter_$=(nudge = 2 | nudge = 3).

VARIABLE LABELS filter_$ 'nudge = 2 | nudge = 3 (FILTER)'.

VALUE LABELS filter_$ 0 'Not Selected' 1 'Selected'.

FORMATS filter_$ (f1.0).

FILTER BY filter_$.

EXECUTE.

CROSSTABS

/TABLES=nudge BY accuracy BY context

/FORMAT=AVALUE TABLES

/STATISTICS=CHISQ CC PHI ETA

/CELLS=COUNT ROW

/COUNT ROUND CELL.

CROSSTABS

/TABLES=coder1 BY coder2

/FORMAT=AVALUE TABLES

/STATISTICS=CHISQ PHI KAPPA

/CELLS=COUNT

/COUNT ROUND CELL.

CROSSTABS

/TABLES=open_ended_coding_final BY accuracy

/FORMAT=AVALUE TABLES

/STATISTICS=CHISQ CC PHI

/CELLS=COUNT ROW COLUMN

/COUNT ROUND CELL.

CROSSTABS

/TABLES=open_ended_coding_final BY context BY nudge

/FORMAT=AVALUE TABLES

/STATISTICS=CHISQ PHI

/CELLS=COUNT ROW COLUMN

/COUNT ROUND CELL.

**OSF link for datasets**

<https://osf.io/mgura/?view_only=0e611cdc9dea4f6d8bf6192fee33e846>
